# Strategies Aimed at Nox4 Oxidase Inhibition Employing Peptides from Nox4 B-Loop and C-Terminus and p22^*phox*^ N-Terminus: An Elusive Target

**DOI:** 10.1155/2013/842827

**Published:** 2013-03-31

**Authors:** Gábor Csányi, Patrick J. Pagano

**Affiliations:** ^1^Vascular Medicine Institute, University of Pittsburgh, 12th Floor BST, 200 Lothrop Street, Pitsburgh, PA 15261, USA; ^2^Department of Pharmacology & Chemical Biology, University of Pittsburgh, 13th Floor BST, 200 Lothrop Street, Pitsburgh, PA 15261, USA

## Abstract

Although NADPH oxidase 4 (Nox4) is the most abundant Nox isoform in systemic vascular endothelial and smooth muscle cells, its function in the vascular tissue is not entirely known. The literature describes a pathophysiological role for Nox4 in cardiovascular disease; however, some studies have reported that it has a protective role. To date, specific Nox4 inhibitors are not available; hence, the development of a pharmacologic tool to assess Nox4's pathophysiological role garners intense interest. In this study, we selected peptides corresponding to regions in the Nox4 oxidase complex critical to holoenzyme activity and postulated their utility as specific competitive inhibitors. Previous studies in our laboratory yielded selective inhibition of Nox2 using this strategy. We postulated that peptides mimicking the Nox4 B-loop and C-terminus and regions on p22^*phox*^ inhibit Nox4 activity. To test our hypothesis, the inhibitory activity of Nox4 B-loop and C-terminal peptides as well as N-terminal p22^*phox*^ peptides was assessed in a reconstituted Nox4 system. Our findings demonstrate that Nox4 inhibition is not achieved by preincubation with this comprehensive array of peptides derived from previously identified active regions. These findings suggest that Nox4 exists in a tightly assembled and active conformation which, unlike other Noxes, cannot be disrupted by conventional means.

## 1. Introduction

NADPH-oxidase- (Nox-) derived reactive oxygen species (ROS) play a central role in the destruction of pathogenic organisms by phagocytes. The phagocyte Nox complex is composed of flavocytochrome b558, an integral membrane heterodimer composed of gp91^*phox*^ (a.k.a. Nox2) and p22^*phox*^, four cytoplasmic protein subunits, p47^*phox*^, p67^*phox*^, p40^*phox*^, and the regulatory low molecular weight GTPase Rac. In resting phagocytes, the Nox enzyme is in a dormant state. Upon activation, the cytosolic Nox subunits translocate to Nox2 and p22^*phox*^ followed by the transfer of one electron from NADPH to molecular oxygen, resulting in the formation of superoxide anion (O_2_^•−^) and microbicidal activity [1]. 

Nox2 is also expressed in cells other than phagocytes, [2, 3] and excessive ROS generation by nonphagocytic Nox2 contributes to a wide variety of disorders [3–5]. Over the past decade since the discovery of Nox2 homologs Nox1, Nox3, Nox4, Nox5, DUOX1 and DUOX2 [4], interest has greatly increased in Nox enzymes and the development of isoform-specific Nox inhibitors. Although numerous chemical compounds have been shown to inhibit Nox enzymes, none of these to our knowledge is specific for one isoform [6, 7]. Importantly, rationally designed, sequence-specific peptide-based inhibitors have the potential to be among the most selective and effective inhibitors of Nox because of their potential to selectively target unique protein interactions within the enzyme. A previous study by our group demonstrated that a peptide sequence mimicking amino acids 86–94 in the first intracellular loop of Nox2 (B-loop) specifically inhibits Nox2 activation *in vitro* [8, 9]. The effectiveness of this peptide to inhibit ROS production *in vivo* has been widely shown, and has led to its wide use in numerous studies [10–15]. To date, peptidic inhibitors have been reserved for Nox2; that is, no prior studies tested whether inhibition of other homologs can be achieved by this strategy.

Nox4 is the most abundant Nox isoform in endothelial cells, vascular smooth muscle cells, and the kidney [16, 17], but it is also expressed in the heart, central nervous system, airways, and skeletal muscle [4]. Animal and human studies have shown that Nox4 plays an important role in the pathophysiology of a wide variety of disorders, including systemic hypertension [18], diabetes mellitus [19], vascular injury [20], atherosclerosis [21], ischemic stroke [22], pulmonary fibrosis [23], and diabetic nephropathy [24]. Collectively, these data suggest that Nox4 oxidase is a major contributor to oxidative stress in these pathologic conditions, and blocking the undesirable actions of Nox4 could become a therapeutic strategy to attenuate oxidative stress in patients with these disorders. Unlike other Nox isoforms, numerous studies have also described that Nox4 is involved in a variety of physiological processes, including cell differentiation, survival, and migration [16, 25–27]. Moreover, a few studies have reported that Nox4 has a protective role in cardiovascular tissue, although this is still somewhat controversial [19, 28, 29]. Presently, no specific inhibitors of Nox4 (small molecule or peptidic) are available to the scientific community [30] to elucidate the pathophysiological and/or physiological roles of Nox4. 

Nox4 oxidase is a unique Nox isozyme as it differs from the usual model of multimeric Nox assembly found in Nox1, Nox2, and Nox3. Indeed, Nox4 does not require interaction with any of the conventional cytosolic Nox subunits for ROS generation and the membrane-bound subunit p22^*phox*^ is, to date, the only known classical subunit associated with Nox4. Recently, Poldip2 has been described as a modulator of Nox4 [31]; however, in this study we aimed to target the core of the enzyme. 

A previous study reported that mutagenesis of arginine residues in the Nox4 B-loop impedes activity of Nox4 [32]. Moreover, it was suggested that the B-loop of Nox4 serves as a binding sequence facilitating interaction of C-terminal NADPH-binding domain of Nox4 with its membrane-spanning region [32]. In addition, deletion of the first 11 amino acids at the p22^*phox*^ N-terminus completely abrogated Nox4 activity [33]. These data suggest that interaction of Nox4 B-loop with the C-terminal domain as well as association of Nox4 with the N-terminus of p22^*phox*^ is important for Nox4 activity. We postulated that recombinant excess of Nox4 B-loop and key p22^*phox*^ N-terminal peptide mimics would disrupt intramolecular B-loop-C-terminal and Nox4-p22^*phox*^ interactions, respectively, leading to inhibition of Nox4-derived ROS production. Accordingly, the aim of the present study was to investigate whether targeting the Nox4 B-loop and C-terminal domain with sequential 15-mer and nonamer peptide sequences disrupts their interaction and inhibits Nox4 activity. In the present study, p22^*phox*^ N-terminal tail peptides were also tested for their ability to inhibit Nox4 activity.

## 2. Materials and Methods

Catalase, diphenyleneiodonium chloride (DPI), flavin adenine dinucleotide (FAD), horseradish peroxidase (HRP), and phenylmethanesulfonyl fluoride (PMSF) were purchased from Sigma-Aldrich (St. Louis, MO, USA). Amplex Red was purchased from Invitrogen (Eugene, OR, USA). Protease inhibitor cocktail was purchased from Roche Diagnostics GmbH (Mannheim, Germany). All peptides were synthesized by the Tufts University Core Facility (Boston, MA, USA). The purity of peptides was over 97%. Since the current studies were carried out using COS-Nox4 cell lysates, it is important to note that the peptides used in this study did not require chimeric design containing *tat* peptide for cell permeation. 

### 2.1. Cell Lines and Cell Culture

COS-22 cells (COS-7 cells stably expressing human p22^*phox*^) were kindly provided by Dr. Mary C. Dinauer (Indiana University, School of Medicine) [34]. COS-22 cells were maintained in Dulbecco's Modified Eagle Medium (Mediatech, Inc., Manassas, VA, USA) with 4.5 g/L glucose, L-glutamine, and sodium pyruvate containing 10% heat-inactivated fetal bovine serum (FBS), 100 units/mL penicillin, and 100 *μ*g/mL streptomycin. 

### 2.2. Plasmid Preparation, Amplification, and Purification

Plasmid encoding full-length human cDNAs for Nox4 (pcDNA3-hNox4) was kindly provided by Dr. David Lambeth (Emory University, GA) [35]. For human Nox4 expression, the BglII/NotI restriction fragment from the pcDNA3-hNox4 was subcloned into the plasmid pcDNA3.1/Hygro(−) (Invitrogen, Carlsbad, CA) to generate pcDNA3.1/Hygro-hNox4. The fragment sequence, in-frame insertion, and orientation were validated by DNA sequencing after PCR amplification. pcDNA3.1/Hygro-hNox4 was amplified into *Escherichia coli* strain TOP10 (Invitrogen, Carlsbad, CA) and purified with a QIA filter plasmid purification kit (QIAGEN Inc., Valencia, CA).

### 2.3. Transfection

Cell transfection was carried out using Lipofectamine LTX and Plus reagent (Invitrogen, Carlsbad, CA, USA), according to the manufacturer's instructions. COS-22 cells were transiently transfected with pcDNA3.1/Hygro-hNox4 (COS-Nox4 cells). Western blot experiments were performed to validate the expression of Nox4 as we reported previously [8]. Twenty-four hours after transfection, cells were harvested by incubating with 0.05% trypsin/EDTA for 5 min at 37°C. Following addition of DMEM/10% FBS to neutralize the trypsin, the cells were pelleted by centrifugation at 1000 ×g for 5 min at 4°C and used for the experiments. 

### 2.4. Hydrogen Peroxide- (H_2_O_2_-) Generating Activity

H_2_O_2_ production was quantified in lysed COS-Nox4 cells as described previously [36]. It is important to note that COS-Nox4 cells do not produce O_2_^•−^ [8]. COS-Nox4 and COS-22 cells were suspended to a concentration of 5 × 10^7^ cells/mL in ice-cold disruption buffer (PBS containing 0.1 mM EDTA, 10% glycerol, protease inhibitor cocktail, and 0.1 mM PMSF). The cells were lysed by five freeze/thaw cycles and passed through a 30-gauge needle five times to further lyse the cells. Throughout all these procedures, extreme care was taken to maintain the lysate at a temperature close to 0°C. Incubation of COS-Nox4 cell lysate (10 *μ*g/100 *μ*L) with peptides was performed in assay buffer (25 mM Hepes, pH 7.4, containing 120 mM NaCl, 3 mM KCl, 1 mM MgCl_2_, 25 *μ*M FAD, 0.1 mM Amplex Red, and 0.32 U/mL of HRP) for 15 or 60 min at room temperature on an orbital shaker (120 movements/min), before the addition of 36 *μ*M NADPH, to initiate H_2_O_2_ production. This relatively low concentration of NADPH was used because it was found that higher concentrations interfered with Amplex Red fluorescence. Fluorescence measurements were made using a Biotek Synergy 4 Hybrid Multi-Mode Microplate Reader (excitation wavelength: 560 nm; emission wavelength: 590 nm). A standard curve of known H_2_O_2_ concentrations was developed using the Amplex Red assay (as per the manufacturer's instructions) and was used to quantify H_2_O_2_ production in lysed COS-Nox4 cells. The reaction was monitored at room temperature for 40 min. The emission increase was linear during this interval. The rate of H_2_O_2_ production was interpolated from the standard curve and is 609 ± 0.01 nmol H_2_O_2_/min/mg protein. The effect of a peptide on Nox4-derived H_2_O_2_ production was expressed as percent inhibition of Nox4, which was calculated by considering H_2_O_2_ production by control mixtures in the absence of peptide as 100%.

### 2.5. Statistical Analysis

All results are expressed as means ± SEM. Significance of the differences was assessed by two-way ANOVA followed by Bonferroni post hoc test. *P* < 0.05 was considered to be statistically significant. 

## 3. Results

### 3.1. Nox4 Catalytic Activity Is Not Inhibited by Preincubation with Nox4 B-Loop Peptides

**Figure 1:**
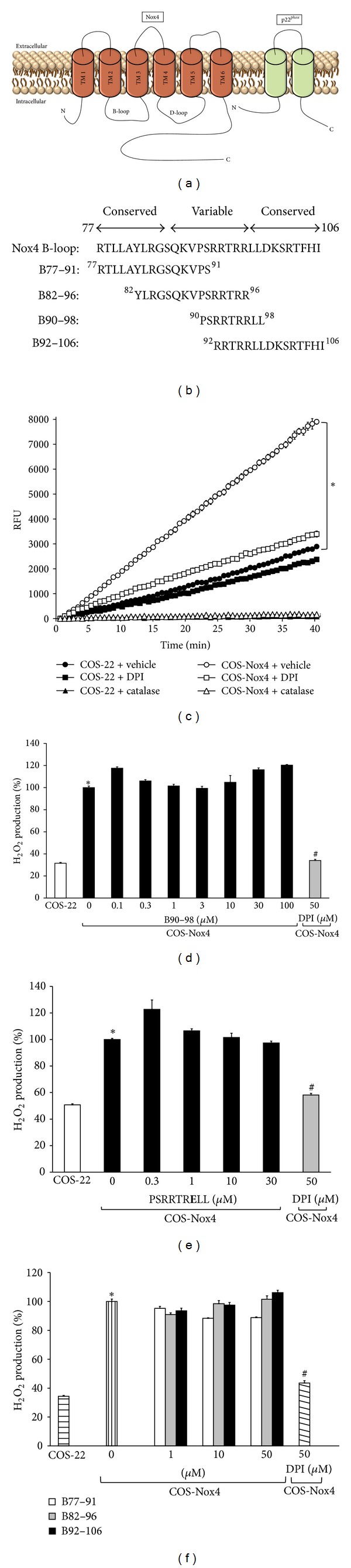
Nox4 B-loop peptides and Nox4 activity. (a) A simplified model of Nox4 oxidase with B- and D-loops located intracellularly. p22^*phox*^, the only known protein required for Nox4 activity, is also shown. (b) Amino acid sequence of Nox4 B-loop and list of overlapping Nox4 B-loop region synthetic 15-mer and nonamer peptides used to target Nox4 activity. The numbers at the N- and C-terminus of each peptide indicate the location of the corresponding residues in the amino acid sequence of Nox4. (c) H_2_O_2_ generated by Nox4 was measured using Amplex Red fluorescence. H_2_O_2_ production was initiated by the addition of 36 *μ*M NADPH. The reaction was monitored at 24°C for 40 min. The flavin-containing enzyme inhibitor diphenyleneiodonium (DPI; 50 *μ*M) was used as a positive control. Identification of H_2_O_2_ was confirmed by inhibition of fluorescence with catalase (3000 U/mL). For comparison, H_2_O_2_ production in nontransfected COS-22 cell lysate is shown. Data represent the mean ± SEM of 3 experiments. *P < 0.05 indicates significant difference between COS-22 and COS-Nox4 cell lysates. (d) COS-Nox4 cell lysates were preincubated with various concentrations of B90–98 (from 0.1 to 100 *μ*M) for 15 min at 24°C, and H_2_O_2_ was measured using Amplex Red fluorescence. Data represent the mean ± SEM of 3 experiments. (e) COS-Nox4 cell lysates were preincubated with the R96E mutant version of B90–98 (PSRRTR**E**LL; 0.3 to 30 *μ*M) for 15 min at 24°C, and H_2_O_2_ was measured. Data represent the mean ± SEM of 3 experiments. (f) COS-Nox4 cell lysates were preincubated with peptides from N- and C-terminal ends of the Nox4 B-loop (B77–91, B82–96, and B92–106; 1 to 50 *μ*M) for 15 min at 24°C, and H_2_O_2_ was measured. Data represent the means ± SEM of 3 experiments. *P < 0.05 indicates a significant difference between COS-22 and COS-Nox4 cell lysates. ^#^ *P* < 0.05 indicates a significant difference between COS-Nox4 + 0 *μ*M peptide and COS-Nox4 + DPI.

The first intracellular loop of Nox4 (B-loop) is essential for catalytic activity, that is, hydrogen peroxide (H_2_O_2_) generation, as previously demonstrated by point mutations targeting this region (Figure 1(a)) [32]. Amino acid sequence alignment revealed three subregions of Nox B-loops where two conserved regions flank a highly variable central region (Figure 1(b)). The two flanking conserved regions are shared by all Nox isoforms, whereas the central region is variable across Nox isoforms [32]. Previous data indicated that B-loop peptides bind to the C-terminal of the enzyme and that this interaction is important for activity [32]. We postulated then that this interaction would be competitively blocked by peptides derived from the B-loop, as we showed for Nox2 [8]. In the current study, peptide sequences derived from the variable and conserved regions of Nox4 B-loop were tested for their ability to inhibit Nox4 activity. As shown in Figure 1(c), the rate of H_2_O_2_ generation was significantly higher in COS-Nox4 cell lysates as compared with nontransfected controls, and ~90% of the Nox4-dependent activity was inhibited by the flavin-containing enzyme inhibitor diphenyleneiodonium (DPI; 50 *μ*M). Detection of H_2_O_2_ was confirmed by inhibition of fluorescence with catalase (3000 U/mL). As demonstrated in Figure 1(d), peptide B90–98 (0.1–100 *μ*M) did not inhibit Nox4-derived H_2_O_2_ production. As substitution of Arg-96 to glutamic acid previously resulted in almost complete loss of Nox4 activity [32], we tested whether the R96E mutant version of B90–98 (PSRRTR**E**LL) is capable of inhibiting Nox4 activity. Similar to the wild-type peptide, the mutant peptide did not inhibit Nox4 activity (Figure 1(e)).

Mutation of Arg-84 to alanine on the N-terminal conserved Nox4 B-loop region [32] and replacement of Ser-101 with acidic residues in the C-terminal Nox4 B-loop conserved region were previously shown to inhibit Nox4 [37]. Thus, we tested whether inhibition of Nox4 activity can be achieved by proximal and distal Nox4 B-loop peptides. Our data demonstrated that preincubation of COS-Nox4 cell lysates with neither peptides B77–91, B82–96, nor B92–106 inhibited H_2_O_2_ production (Figure 1(f)). 

### 3.2. Nox4 C-Terminal Tail Peptides Do Not Inhibit Nox4 NADPH Oxidase

**Figure 2:**
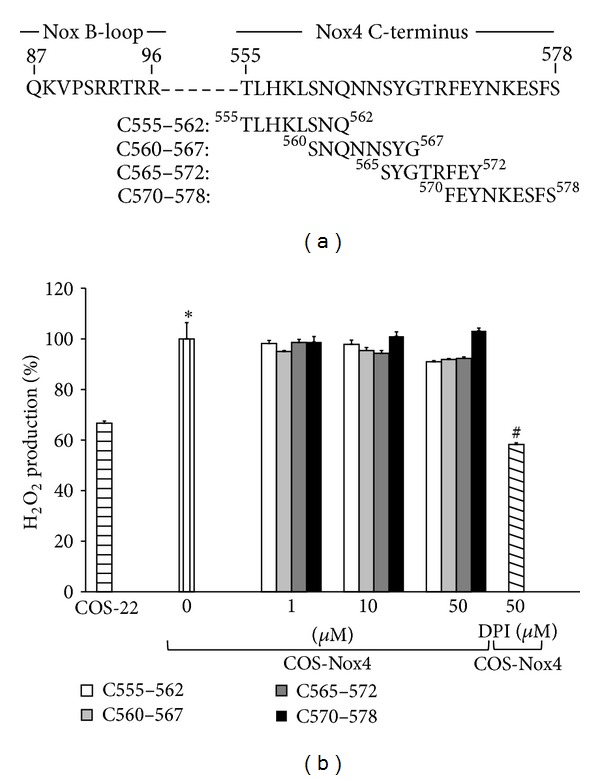
Examination of Nox4 C-terminal tail peptides as Nox4 inhibitors. (a) Map of overlapping Nox4 C-terminal region synthetic octa- and nonapeptides used to inhibit Nox4 activity. Numbers correspond to location and span of the N- and C-terminus for each peptide. (b) COS-Nox4 cell lysates were preincubated with various concentrations of C555–562, C560–567, C565–572, and C570–578 for 15 min at 24°C, and H_2_O_2_ was measured using Amplex Red. Data represent the mean ± SEM of 3 experiments. *P < 0.05 indicates a significant difference between COS-22 and COS-Nox4 cell lysates. ^#^ *P* < 0.05 indicates a significant difference between COS-Nox4 + 0 *μ*M peptide and COS-Nox4 + DPI.

A previous study demonstrated that the last 22 amino acids of the whole Nox4 protein are critical for catalytic activity (Figure 2(a)) [38]. The presence of charged residues in this flexible region of Nox4 may suggest that electrostatic effects could promote interaction between the C-terminus and B-loop and/or p22^*phox*^. Thus, three overlapping octameric peptides (C555–562, C560–567, and C565–572) and a nonameric peptide (C570–578) were synthesized to cover the last 22 amino acids of Nox4, and these were tested for their ability to inhibit Nox4 activity. Application of these C-terminal tail peptides did not affect Nox4 activity (Figure 2(b)). 

### 3.3. p22^*phox*^ N-Terminal Tail Peptides Do Not Inhibit Nox4 NADPH Oxidase

**Figure 3:**
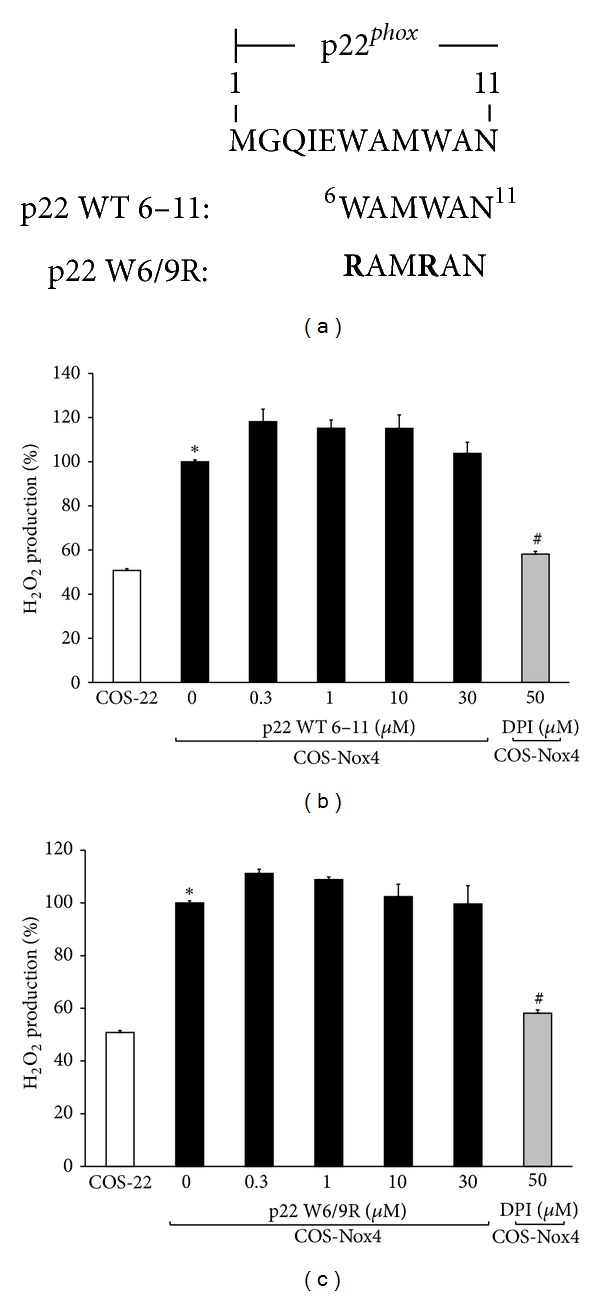
Testing whether p22^*phox*^ N-terminal tail peptides inhibit Nox4 activity. (a) The first 11 amino acids of p22^*phox*^ are displayed. The peptide sequence of p22^*phox*^ between amino acids 6 and 11 is shown (p22 WT 6–11; WAMWAN); point mutations of W6/9R (p22 W6/9R; **R**AM**R**AN) are indicated in boldface type. The numbers at the N- and C-terminus of WAMWAN indicate the location of the corresponding residues in the amino acid sequence of p22^*phox*^. (b) COS-Nox4 cell lysates were preincubated with various concentrations of p22 WT 6–11 for 15 min at 24°C, and H_2_O_2_ was measured using Amplex Red. Data represent the mean ± SEM of 3 experiments. (c) COS-Nox4 cell lysates were preincubated with various concentrations of p22 W6/9R for 15 min at 24°C, and H_2_O_2_ was measured using Amplex Red. Data represent the mean ± SEM of 3 experiments. *P < 0.05 indicates a significant difference between COS-22 and COS-Nox4 cell lysates. ^#^ *P* < 0.05 indicates a significant difference between COS-Nox4 + 0 *μ*M peptide and COS-Nox4 + DPI.

Expression of p22^*phox*^ is required for Nox4 activity, and, to date, p22^*phox*^ is the only known classical core protein associated with Nox4 [39]. Mutagenesis studies provided evidence that deletion of a large span of the p22^*phox*^ C-terminus (terminal 130 amino acids) did not affect Nox4 activity [33]. In contrast, deletion of the first 11 amino acids at the p22^*phox*^ N-terminus attenuated Nox4 activity. Deletion of the first 5 amino acids did not affect Nox4 activity, suggesting that the N-terminal region of p22^*phox*^ between amino acids 6 and 11 is sensitive to modification. A peptide sequence between amino acids 6 and 11 (p22 WT 6–11 (WAMWAN)) was thus synthesized and tested for its ability to inhibit Nox4 (Figure 3(a)). As demonstrated in Figure 3(b), peptide p22 WT 6–11 did not inhibit Nox4 activity. Mutation of tryptophans within this sequence to arginine (p22 W6/9R (RAMRAN)) also abolished Nox4 activity [33]. Similar to the native peptide, the p22 W6/9R mutant did not inhibit Nox4-derived H_2_O_2_ production. 

### 3.4. Increased Incubation Time and Temperature Do Not Facilitate Peptide-Induced Inhibition

Our data demonstrate that preincubation of COS-Nox4 cell lysates with Nox4 B-loop, Nox4 C-terminal tail, and N-terminal p22^*phox*^ peptides for 15 min at 24°C did not inhibit Nox4 activity. To allow more time for the peptides to disrupt the targeted domain interactions, we increased incubation time up to 60 min. Our results demonstrated that none of the Nox4 B-loop, Nox4 C-terminal tail, and N-terminal p22^*phox*^ peptides inhibit Nox4 activity after 60 min of incubation (data not shown). Moving to a new approach, we tested whether providing more kinetic energy, which we proposed to be more favorable for peptide interference with the tightly assembled conformation of Nox4, would facilitate peptide-induced inhibition. Thus, in an attempt to induce temporary perturbations in the structure of Nox4, these experiments were also performed at 37°C. Again, no inhibition of Nox4 was achieved (data not shown).

## 4. Discussion

This is the first study to our knowledge that seeks to inhibit Nox4 using a peptidic strategy. Indeed, synthetic peptides mimicking key residues in Nox2, p22^*phox*^, p47^*phox*^, and Rac1 have been shown to interfere with the activation process of Nox2 and inhibit Nox2-derived O_2_^•−^ production [8, 9, 40–43]. Those studies identified domains of functional importance in the assembly of Nox2 oxidase and provided key information about the transformation of the enzyme complex from the dormant, inactive conformation to its active state of the enzyme. Nox1, Nox3, and Duox require cytosolic subunits for activation, while Nox5 does not. Nox4, with p22^*phox*^, appears to constitutively generate H_2_O_2_ without the requirement of activating cytosolic subunits, with the exception of Poldip2 [31]. With a desire to target the core of the enzyme, the present study was designed to (a) target multiple residues in the Nox4 sequence with the purpose of determining whether prior information gleaned from mutational studies [32, 33] translates to the potential for peptide mimics disrupting key intramolecular interactions for Nox4 activity and (b) develop isoform-specific peptidic Nox4 inhibitors using such findings. 

Previous studies showed that multiple residues in the B-loop and the C-terminal end are critical for the catalytic activity of Nox4 [32, 37]. A recent study using fluorescence polarization demonstrated binding between the Nox4 B-loop and Nox4 dehydrogenase domain and showed that this interaction is weakened by mutation of arginine residues in the B-loop variable region [32]. With this in mind, in the current study, we tested whether application of a peptide mimic (B90–98) from the variable region of Nox4 B-loop interferes with the activity of Nox4. An important premise for this work was our previous findings illustrating that mimicking the corresponding B-loop region in Nox2, known as Nox2ds, selectively and potently inhibits Nox2-derived O_2_^•−^ production [8]. We went on to provide evidence that Nox2ds, but not its scrambled control, acts as a competitive inhibitor of the enzyme. The theory then for this study was that these B-loop peptides would likewise act as competitive inhibitors of previously proposed key intramolecular interactions occurring between domains of Nox4. Surprisingly, peptide B90–98 up to 100 *μ*M did not inhibit Nox4-derived H_2_O_2_ production as measured by Amplex Red fluorescence. Incidentally, Amplex Red was chosen as the most logical method for detecting H_2_O_2_, which is widely considered the primary Nox4 product [44]. 

Next, in the interest of pursuing a comprehensive approach to these studies, we progressed to testing whether peptides derived from the conserved regions of Nox4 B-loop were capable of inhibiting Nox4. We found that incubation of COS-Nox4 cell lysates with mimics of the N- and C-terminal conserved B-loop regions (B77–91, B82–96, and B92–106) did not inhibit activity. With these results, we postulated that a B-loop peptide mimic might instead be replicating a normal intrinsic function of the B-loop. For this reason, we tested whether peptide B90–98 mutated at residue 96 (R96E), which previously was identified to be critical for Nox4 activity [32], inhibits Nox4-derived H_2_O_2_ production. We postulated that this mutated peptide could act as a dominant negative in that regard. Similar to the wild-type peptide, the mutant peptide did not inhibit Nox4-derived H_2_O_2_ production. One possible explanation for the lack of effect could have been that a multidimensional intramolecular interaction of the enzyme is at play, and thus targeting only one region could be insufficient to interfere with enzymatic activity. A second possibility is that Nox4 exists in a tightly assembled conformation and its unique tertiary structure permits an active electron transferring arrangement that cannot be disrupted by targeting discrete binding sites with either the native or mutant peptide. 

A previous study demonstrated that a C-terminal region downstream of the NADPH-binding motif is important for Nox4, but not Nox2, activity [38]. With further analysis, von Löhneysen et al. identified the last 22 amino acids of Nox4 as essential to activity of the isozyme. Based on these data, we postulated that small peptides targeting this more flexible region would inhibit Nox4 activity. To test the hypothesis, sequential octameric and nonameric peptides were synthesized to encompass the last 22 amino acids of Nox4 and tested for their ability to inhibit Nox4. Likewise, our data demonstrate that none of these C-terminal tail peptides inhibited Nox4 activity. Taken together, these data appear to suggest that Nox4 exists in a tightly assembled, active conformation, thus explaining why peptides targeting intramolecular interactions of the enzyme are not able to interfere with its activity. This observation would be consistent with the reported constitutive and high capacity Nox4 activity. 

Shifting to a new approach, we tested whether small peptides targeting the Nox4-p22^*phox*^ intermolecular interaction could inhibit Nox4. A previous study showed that a peptide (175–194) derived from p22^*phox*^ inhibits ROS production by Nox2 [42]. Subsequent studies by Dahan et al. using peptide walking identified domains throughout the p22^*phox*^ protein sequence (9–23, 31–45, 47–61, 85–99, and 113–127) that are important for Nox2 activity [45]. It is, however, completely unknown whether introduction of p22^*phox*^ peptides can disrupt Nox4-p22^*phox*^ interaction and inhibit Nox4-derived H_2_O_2_ production. Importantly, a large part of the p22^*phox*^ C-terminus does not seem to be important for Nox4 activity as deletion of amino acids up to and including the last 130 amino acids does not affect Nox4 activity [33]. Thus, targeting the C-terminal domain of p22^*phox*^ is not likely to inhibit Nox4 activity. In contrast, deletion of the first 11, but not the first 5, amino acids at the p22^*phox*^ N-terminus reportedly abolished Nox4-derived H_2_O_2_ production [33]. This suggested to us that the N-terminal region of p22^*phox*^ between amino acids 6 and 11 might be sensitive to intervention. We targeted this region using a peptide sequence between amino acids 6 and 11 (p22 WT 6–11 (WAMWAN)) and measured Nox4-derived H_2_O_2_ production. Once again, the data demonstrated that p22 WT 6–11 did not inhibit Nox4 activity. We next considered previous work showing that mutation of tryptophans within this region to charged residues, such as arginine, abolished Nox4 activity [33]. Thus, in an attempt to mimic this inactive catalytic site, we tested whether the W6/9R mutant version of p22 6-11 (p22 W6/9R) could inhibit Nox4 activity. Similar to the wild-type peptide, the mutant peptide did not inhibit Nox4-derived H_2_O_2_ production. 

As we observed, preincubation of COS-Nox4 cell lysates with Nox4 B-loop, Nox4 C-terminal tail, and N-terminal p22^*phox*^ peptides for 15 min at 24°C did not inhibit H_2_O_2_ production. To allow more time for the peptides to disrupt the targeted domain interactions, the incubation time was increased up to 60 min. Notably, previous studies illustrate that 60 min is more than sufficient to inhibit Nox activity [40]. In our hands, 60 min incubation did not reveal inhibitory activity. To go one step further, these experiments were performed at 37°C in an attempt to increase access and likelihood of interaction of peptides with the Nox4 complex. Again, no inhibition of Nox4 was achieved using these peptides. Our data suggest that alternative strategies to improve access or penetrability of peptides into the Nox4-p22^*phox*^ complex may be necessary to achieve inhibition of Nox4. 

In conclusion, our findings suggest that the Nox4-p22^*phox*^ complex is unperturbed by a wide array of rationally selected peptide mimics. This is not to say that other to-date unidentified active regions of the enzyme could not eventually be devised as inhibitors. Moreover, a more comprehensive study using peptide walking of the entire Nox4 protein could be warranted. It is also plausible that various combinations of peptide mimics may be effective. This is currently an area of active investigation in our laboratory. That notwithstanding, it is our tentative conclusion that the tightly assembled Nox4 complex creates a formidable barrier to peptidic interference and thus greater access could be viewed as the *sine qua non* of these strategies. In that vein, we are actively pursuing other strategies to temporarily improve access (unfolding) or penetrability of peptides into the Nox4 complex. Application of peptidic inhibitors targeting domain interactions of Nox4 while in the endoplasmic reticulum (before its native folding is complete or associates with p22^*phox*^) may be another viable strategy. Of course, small molecule inhibitors that specifically target the above-identified interactions are likely to elude limitations of access and are currently a focus of intense interest in our laboratory.
